# Temporal Dynamics in Rumen Bacterial Community Composition of Finishing Steers during an Adaptation Period of Three Months

**DOI:** 10.3390/microorganisms7100410

**Published:** 2019-10-01

**Authors:** Qinghua Qiu, Chaoyu Gao, Zhibiao Gao, Muhammad Aziz ur Rahman, Yang He, Binghai Cao, Huawei Su

**Affiliations:** 1 State Key Laboratory of Animal Nutrition, College of Animal Science and Technology, China Agricultural University, Beijing 100193, China; rcauqqh@cau.edu.cn (Q.Q.);; 2 Institute of Animal and Dairy Sciences, University of Agriculture, Faisalabad 35200, Pakistan

**Keywords:** dynamic variation, finishing steer, rumen bacterial community, sampling frequency

## Abstract

The objective of this study was to explore whether collecting rumen samples of finishing steers at monthly intervals differed, and whether this difference or similarity varied with diets. For these purposes, 12 Chinese Holstein steers were equally divided into two groups. The dietary treatments were either standard energy and standard protein (C) or low energy and low protein (L). Rumen samples were obtained on day 30, day 60 and day 90 from both dietary treatments and were analyzed by using 16S rRNA gene sequencing. The results showed that monthly intervals had no effect on the richness and evenness of the rumen bacterial community in the two diets. However, taxonomic difference analysis (relative abundance >0.5%) revealed that the relative abundance of three phyla (*Proteobacteria*, *Fibrobacteres* and *Cyanobacteria*) and six genera (*Rikenellaceae_RC9_gut_group*, *Ruminococcaceae_NK4A214_group*, *Fibrobacter*, *Eubacterium_coprostanoligenes_group*, *Ruminococcaceae_UCG-010* and *Ruminobacter*) were significantly different between monthly sampling intervals, and the difference was prominent between sampling in the first month and the subsequent two months. Moreover, the differences in abundances of phyla and genera between monthly sampling intervals were affected by diets. Analysis of similarity (ANOSIM) showed no significant differences between monthly sampling intervals in the C diet. However, ANOSIM results revealed that significant differences between the first month and second month and between the first month and third month were present in the L diet. These results indicated that temporal dynamics in rumen bacterial community composition did occur even after an adaptation period of three months. This study tracked the changes in rumen bacterial populations of finishing cattle after a shift in diet with the passage of time. This study may provide insight into bacterial adaptation time to dietary transition in finishing steers.

## 1. Introduction

The complex microbiota in the rumen including bacteria, protozoa, archaea, and fungi contributes to the unique function of ruminants in transforming plant polymers and compounds to useable nutrients [1]. The rumen provides a favorable environment for microbes to grow, and these microbes ferment feedstuffs to produce volatile fatty acids and microbial protein, which are major sources of nutrition for the maintenance, growth or production of ruminants [2]. In this unique and mutually beneficial host-microbiome interaction, the ability of ruminants to digest feeds is maximized by its coevolved microbiome [3]. Many reports have indicated that the structure of the rumen microbial community is associated with host productivity [4,5,6]. Therefore, improvement in productivity of ruminants could be obtained by better understanding the relationship between the rumen microbial community and host productivity.

Rumen bacteria are the most abundant microbiota in terms of diversity and contribute about 95% of the microbial community [7,8]. Therefore, investigating the composition of rumen bacteria could be a typical approach for exploring the relationships between rumen microbiota and host productivity, and obtaining representative rumen samples is the first step. Numerous studies had reported that the rumen sampling interval (hours, days, weeks and years) may influence the rumen microbial community composition [9,10]. Li et al. (2009) reported no differences in bacterial composition within three hours before feeding, and three and nine hours after feeding. Weimer et al. (2010, 2017) reported that it required several weeks for bacterial composition to return to the cows’ original bacterial structure after an exchange of ruminal contents and the return time varied with individuals. Individual animal variability was also observed by Mohammed et al. (2012) [11] and Zhou et al. (2018) [12]. Machado et al. (2016) [13] reported an adaptation period of two weeks for bacterial community composition to stabilize on a new diet. Noel et al. (2017) [14] observed similar bacterial communities in grazing cattle and found that the small change between seasons in rumen bacterial communities occurred due to diet. Moreover, parity may or may not influence rumen bacterial community, as Jewell et al. (2015) [15] found high similarities between the first and second lactation cycles in terms of the bacterial community, whereas Pitta et al. (2014) reported considerable differences between parities. Recently, Clemmons et al. (2019) [16] reported that growing steers did not reach a stable bacterial community before an adaptation period of eight weeks. These reports have provided valuable insights into host specificity and the adaptation period to dietary transition. However, the required time for the bacterial community composition in finishing cattle to stabilize from a change in diet is still unknown.

It is well known that diet could impact the structure of the rumen microbiome [1,17] and may influence the time to acclimate to dietary transition [18]. Anderson et al. (2016) found that rumen bacteria adapted faster to a diet with high concentrate. Less time for stabilization of a bacterial would improve productivity, because shifts in bacterial composition are associated with milk fat depression [19] and stable microbiomes improve host health [16]. Therefore, stabilization time would be another consideration for optimizing diet.

In general, the fattening period of the Chinese beef cattle production system is over six months [20,21], and is divided into two or three phases, with each phase lasting approximately three months. Examining the same animals across different months provides the opportunity to explore the fluctuation or stability of the bacterial community under production conditions [14]. Therefore, it is of great significance to explore how the bacterial community in the rumen changes as fattening months advance and in what manner the changes occur on different diets. We hypothesized that the bacterial community composition in the rumen would stabilize before three months, but the stabilization time or fluctuation may vary with diets.

## 2. Materials and Methods

### 2.1. Ethics Statement

Animal care and experimental procedures were handled strictly in accordance with the recommendations in the Guide for the Care and Use of Laboratory Animals of the National Institutes of Health of China. The experimental protocols were approved by the China Agricultural University Animal Care and Use Committee (Permit No. AW09059102-2, 5 September, 2017).

### 2.2. Experimental Design and Sample Collection

Twelve Chinese Holstein steers with body weight 467 ± 34 kg and age 14 ± 0.5 months were selected from a commercial beef cattle farm and randomly assigned to one of the two dietary treatments: Standard energy and standard protein (metabolizable energy (ME) = 2.53 Mcal/kg, crude protein (CP) = 11.9%; C) and low energy and low protein (ME = 2.35 Mcal/kg, CP = 10.5%; L). These two diets are mostly applied in the early fattening stage of cattle in China. All animals were fed the same pre-experimental diet (Table 1) for four months before the start of dietary treatments. During the pre-experimental and experimental period, management and environmental condition were the same for all steers. Monensin was added in the pre-experimental diet and supplementation of monensin was continued until the end of the experiment. The duration of the experiment was three months, and each month was considered as an individual period. In the C diet, each individual period was designated as C1, C2 and C3, while in the L diet each individual period was designated as L1, L2 and L3. The experimental feed ingredients profile and nutrient composition are presented in Table 1. Steers were fed twice daily at 7:30 and 16:30. Animals were fed ad libitum and refusal was ensured at 5%. Fresh clean water was available to the animals round the clock. Dry matter intake (DMI) was recorded daily, and the temperature and humidity were recorded every 15 min throughout the trial using a temperature and humidity recorder (Meacon, Hangzhou, China).

Three hours after morning feeding on days 30, 60 and 90, approximately 40 mL of rumen sample was obtained using esophageal tubing as described by Paz et al. (2016) [22], and both solid and liquid fractions were collected. Additionally, the device was rinsed thoroughly with clean water between sample collections to prevent cross contamination between individuals, and the first fraction was discarded to minimize saliva contamination. In addition, 2.0 mL of the rumen samples were repacked into cryogenic vials (Corning Incorporated, New York, NY, USA) immediately after collection and were stored in liquid nitrogen before DNA extraction.

### 2.3. DNA Extraction

The DNA of rumen samples (both solid and liquid fractions) was isolated using an OMEGA Stool DNA Kit (Omega Bio-Tek, Norcross, GA, USA) following the manual instructions with some modifications as follows: The two-step of bead-beating was performed using a homogenizer and a water incubation at 95 °C for 5 min was operated between the two bead-beating steps, which is very similar to Paz et al. (2016). The purity and quality of the genomic DNA were evaluated on a 1% agarose gel, combining with determination by a NanoDrop spectrophotometer (NanoDrop 2000 Technologies Inc., Wilmington, DE, USA). Furthermore, all obtained DNA was stored at −80 °C before further analysis.

### 2.4. PCR Amplification and MiSeq Sequencing

A total of 30 rumen samples, randomly selected 5 steers from each period on each diet according to body weight, were extracted for DNA. Therefore, a total of 30 (15 for both the C and L diet groups, Table S1) high-purity and quality genomic DNA samples were delivered to the Allwegene Company (Beijing) for PCR amplification and MiSeq sequencing. The V3 to V4 hypervariable region, as recommended for exploring the bacterial community by Sinclair et al. (2015) [23], of the bacterial 16S rRNA genes were amplified, and the barcoded primers were the same as our previous study [20]. A well-optimized 25 μL reaction system was established as follows: 12.5 μL of KAPA 2G Robust Hot Start Ready Mix, 1 μL of forward primer (5 μM), 1 μL of reverse primer (5 μM), 5 μL of template DNA, and 5.5 μL of ddH_2_O. The PCR conditions included an initial incubation at 95 °C for 5 min; followed by 32 cycles of 95 °C for 45 s, 55 °C for 50 s, and 72 °C for 45 s; and a final extension step at 72 °C for 10 min. Each sample was amplified in triplicate, and three PCR products per sample were mixed together to reduce reaction-level PCR biases. PCR products were checked on a 2% agarose gel and purified by an AxyPrep DNA Gel Extraction kit (Axygen Biosciences, Union City, CA, USA). Purified products were quantified using the Quanti-Fluor™-ST system (Promega, Madison, WI, USA). Paired-end sequencing was performed by means of the Illumina MiSeq platform (San Diego, CA, USA) following the manufacturer’s instructions.

### 2.5. Sequencing Analysis

Sequencing data were analyzed using the quantitative insights into microbial ecology (QIIME) version 1.9.1 (https://qiime.org/). Paired end reads were merged by Fast Length Adjustment of Short reads (FLASH, version 1.2.11, http://ccb.jhu.edu/software/FLASH/) [24] with a minimum overlap of 10 bp and the maximum mismatch rate of 0.10. Sequences were filtered if they met one of the following criteria: Shorter than 260 bp, contained ambiguous bases or chimeric sequences, low quality score of below 20, a mismatch to primer sequences or barcode tags. The high-quality sequences were clustered into operational taxonomic units (OTUs) at a similarity level of 97% using the UPARSE pipeline (USEARCH v11.0.667, http://www.drive5.com/usearch/) [25]. OTUs across all samples were rarefied to the lowest sample depth (41,070 reads) based on the pseudo-random number generator of QIIME.

Alpha diversity indexes, including Chao1, Good’s coverage, observed species, phylogenetic diversity (PD) whole tree and Shannon index, were calculated using Mothur [26] version 1.39.5 (Patrick Schloss, Ann Arbor, USA). 

Taxonomy classifications for each OTU were obtained by assigning against the Silva bacterial alignment database version 132 [27], which adopted the Ribosomal Database Project (RDP) classifier (http://sourceforge.net/projects/rdp-classifier/) with a confidence threshold of 70% [28].

Beta diversity was presented using Principal Coordinate Analysis (PCoA) and the PCoA was based on weighted UniFrac distances using the GUniFrac packages (https://cran.r-project.org/web/packages/GUniFrac/index.html; author, Jun Chen; published 2018; version 1.1). Analysis of similarity (ANOSIM) was taken to test the grouping differences of each diet separately in the PCoA plot with 999 permutations using the vegan package (https://cran.r-project.org/web/packages/vegan/index.html; author, Jari Oksanen et al.; published 2019; version 2.5-5). Pearson correlation coefficients (r) and FDR corrected values (q) were calculated using the Psych packages (https://cran.r-project.org/web/packages/psych/; author, William Revelle; published 2019; version 1.8.12), to display correlations between DMI, temperature or humidity and given genera. The heatmap was performed using GraphPad Prism (version 8.0.2 (263), GraphPad Software, Inc., San Diego, CA).

The raw sequences used in this study were stored on the Sequence Read Archive (SRA) of NCBI, and the SRA accession number is PRJNA527936.

### 2.6. Statistical Analysis

For alpha diversity indexes, the total number of OTUs and a given bacterium at the phylum or genus level (relative abundance >0.5%), repeated measures in generalized linear mixed model procedure of SPSS (version 20, IBM Corporation, Armonk, New York, United States) were taken because all animals repeated in subsequent periods (Table S1). The model included fixed effects for the period and diet, and an animal was considered a random effect. The repeated measures were the sampling periods. Differences among the sampling periods were compared using Tukey tests, and *p* < 0.05 was considered statistically significant, which was marked with different lower-case letters within the same row in tables; a *p* value between 0.05 and 0.10 was regarded as a tendency. Of particular note is that differences in bacterial composition due to individual dietary effects will not be discussed since the objective of this study was to explore sampling periods and whether this variation was affected by diets.

## 3. Results

### 3.1. Temperature, Humidity, DMI and Their Correlations between Genera

During the 90 days of the experimental period, the average temperature was −1.15, −2.80 and 2.96 °C, and the relative humidity was 34.75%, 28.76% and 37.06% in the first, second and third months, respectively. The DMI of the C diet group was 11.11, 11.22 and 11.48 kg/d in the first, second and third months, respectively. The DMI (kg/d) for the L diet group was 10.11, 10.40 and 11.05, for the first, second and third months, respectively.

Correlations between temperature, humidity, DMI and genera are shown in Figure S1. Six genera were observed to be associated with DMI, and two genera with temperature. Of these, *Moryella* was positively (*r* = 0.55, *q* = 0.002) associated with DMI, and *Succinivibrionaceae_UCG-002* and *Ruminobacter* were positively (*r* > 0.35, *q* < 0.05) associated with temperature. No genera with relative abundance great than 0.5% were found to be associated with humidity.

### 3.2. Sequencing Depth and Coverage

After filtering data of low quality, removing short sequences and chimeras, a total of 2,389,150 sequencing reads were obtained from the 30 samples, with a mean of 79,638 sequencing reads for each sample. For the final high-quality sequences, 99.98% were between the lengths of 400 and 440 bp. Shannon–Wiener curves were generated to evaluate whether the sequencing depth was adequate to represent rumen bacteria using Shannon index for all samples. Shannon–Wiener curves (Figure S2) showed that all samples converged, and the increase of the Shannon index tended to be subtle as more reads were sampled, suggesting that the current sequencing depth was sufficient to assess major members of the rumen bacterial composition. The percentage of Good’s coverage indicated that the current sequencing depth could represent at least 97.5% of the bacterial community.

### 3.3. Operational Taxonomic Unit Cluster Analysis

Based on a similarity level of 97%, a total of 1327 and 1543 OTUs were generated in the C and L diet groups, respectively. The sampling interval of months had no significant differences in the total number of OTUs in both the C and L diet groups. Besides, no interactions were observed between diet and the sampling period of the total number of OTUs (Table 2).

### 3.4. Alpha Diversity Analysis

Alpha diversity metrics indicated that the Shannon index, Chao1, observed species and the PD whole tree were similar at monthly sampling intervals in the C and L diet groups, and no interactions were observed between diet and sampling period of these alpha diversity metrics (Table 2).

### 3.5. Taxonomic Analysis

The taxonomic analysis at the phylum level is shown in Table 3. *Bacteroidetes* and *Firmicutes* were the two phyla with the highest relative abundances in both diet groups, whereas no significant differences in abundances of these two phyla were observed among the monthly intervals in both diet groups. *Proteobacteria* was higher (*p* = 0.035) in C3 than that in C1, whereas this phylum showed no significant differences (*p* = 0.783 and 0.112, respectively) between C1 and C2 and C2 and C3. *Fibrobacteres* was higher (*p* = 0.016) in C2 than in C1, and a trend (*p* = 0.080) and similarity (*p* = 0.644) were observed between C1 and C3 and C2 and C3, respectively. *Fibrobacteres* was higher (*p* = 0.018) in L3 than that in L1; however, no significant differences (*p* = 0.349 and 0.211, respectively) were observed between L1 and L2 and L2 and L3. The relative abundance of *Cyanobacteria* increased as months progressed in the C diet group, with similarity (*p* = 0.752) between C2 and C3. In addition, no interactions were observed between diet and sampling intervals of these phyla.

The taxonomic analysis at the genus level is shown in Table 4. *Prevotella* and *Rikenellaceae_RC9_gut_group* were the most predominant genera in both diet groups. *Rikenellaceae_RC9_gut_group* was significantly lower in the last two months than that in the first month in the L diet group. *Ruminococcaceae_NK4A214_group* and *Fibrobacter* were the other two genera with relative abundance greater than 1%, which showed significant differences between sampling intervals. *Ruminococcaceae_NK4A214_group* was higher in the first month than that in the last two months, whereas similarities were observed between the second and third month in both the C and L diet groups. *Fibrobacter* showed higher (*p* = 0.016) relative abundance in C2 than in C1, with a trend (*p* = 0.077) between C1 and C3 and similarity (*p* = 0.649) between C2 and C3. *Fibrobacter* showed a monthly increase with significance (*p* = 0.017) between L1 and L3, but similarities (*p* = 0.343 and 0.210, respectively) between L1 and L2 and L2 and L3. *Eubacterium_coprostanoligenes_group* and *Ruminococcaceae_UCG-010* decreased as months advanced in the L diet group, with similarities (*p* = 0.880 and 0.996, respectively) between L2 and L3. *Ruminobacter* was significantly higher (*p* = 0.009) in C3 than in C1, but there were no significant differences (*p* = 0.433) between C1 and C2 and there was a tendency (*p* = 0.086) between C2 and C3. In addition, no interactions were observed between diet and sampling intervals of these genera.

### 3.6. Beta Diversity

**Figure 1:**
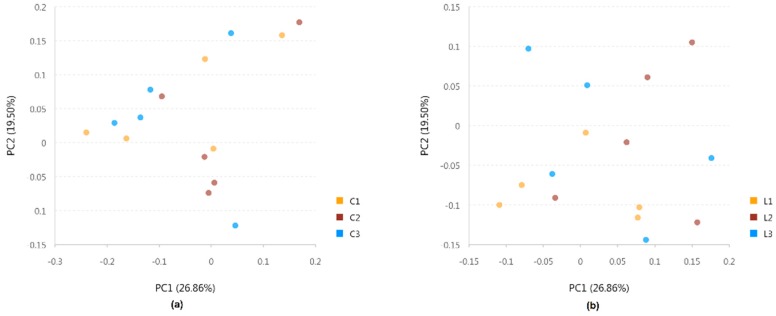
Principal coordinated analysis (PCoA) of rumen bacterial communities based on weighted UniFrac distances in the two diets. (**a**) Standard energy and standard protein (C) C1, C2 and C3 indicate rumen samples collected from the first, second and third month in the C diet group, respectively; (**b**) low energy and low protein (L) L1, L2 and L3 indicate rumen samples collected from the first, second and third month in the L diet group, respectively.

PCoA analysis based on weighted UniFrac metrics was performed to test the differences between sampling intervals separately in the C and L diet groups, represented in Figure 1a,b, respectively. No obvious clusters were observed in the C or L diet groups according to monthly sampling intervals. ANOSIM showed a tendency of difference in rumen bacterial composition between C1 and C3 (*R* = 0.2, *p* = 0.098), whereas no significant differences (*p* = 0.182 and 0.522, respectively) were found between C1 and C2 and C2 and C3. Significant differences were observed between L1 and L2 (*R* = 0.364, *p* = 0.006) and between L1 and L3 (*R* = 0.836, *p* = 0.007). No significant differences were observed between L2 and L3 (*R* = 0.004, *p* = 0.472).

## 4. Discussion

### 4.1. Correlations between Genera and DMI, Humidity and Temperature

*Moryella* has been reported to be core member of ruminal microbiota in diets with faster ruminal passages, such as in non-total mixed rations or high-concentrate diets [29]. A previous study reported that an increased rumen fill of dry matter could shape the rumen bacterial composition by distinctly altering the abundance of fast or slow growing microbes [30]. In the present study, *Moryella* was found to be positively associated with DMI, which could be partly explained by higher DMI increasing the rumen capacity and fill, thus a fast passage being required to ensure proper rumen metabolism. This phenomenon was also observed in the relative abundance of *Moryella* in diets, where a higher abundance (0.86% vs. 0.38%, *p* = 0.004) was found in the C diet than that in the L diet, because a higher concentrate was offered in the C diet. 

*Succinivibrionaceae_UCG-002* and *Ruminobacter* are two genera in the family *Succinivibrionaceae*, and this family was thought to be sensitive to changes in individual, diet and environment [31,32]. In this study, positive correlations were observed between *Succinivibrionaceae_UCG-002* and temperature and between *Ruminobacter* and temperature, which is in agreement with Bach et al. (2019) who found a negative association between *Ruminobacter* and DMI [33], because DMI decreased with an increase in temperature at similar humidity [34]. However, it is strange that no associations were found between any genus and humidity, perhaps the humidity level in the current study was not enough to highlight the differences, but further studies are required to prove this hypothesis.

### 4.2. Differential Evaluation by Alpha Diversity and Beta Diversity

Alpha diversity is used to describe the mean species richness and evenness in a single sample or collection of samples [35]. Alpha diversity metrics showed no significant differences in samples collected at different time intervals of months in the two diets. However, alpha diversity is determined in the absence of information regarding differences between individuals [36]. The PCoA analyses displayed no obvious clusters in the two diets. However, ANOSIM showed significant differences between L1 and L2 and between L1 and L3. Further taxonomic analysis also revealed that significant differences at the phylum and genus levels did exist among monthly sampling intervals in the two diets. These results indicated that a comprehensive assessment based on Alpha and Beta diversity should be applied to evaluate the differences or similarities.

### 4.3. Adaptation Period and Stabilization of the Microbiota

The adaptation period is widely regarded as a vital period to avoid carry-over effects from previous experimental treatments and to potentially promote or impair subsequent performance and health [13,37]. Brown et al. (2006) found that a transitional period of less than 14 days on concentrate levels from 55 to 90% led to a reduction in subsequent growth performance. Swanson et al. (2018) reported that an adaptation period of two weeks or four weeks had little effect on growth performance (e.g., final body weight and average daily gain). However, slight risks were present for feedlot steers with an adaptation period of less than four weeks, mainly for animals suffering from subacute or acute acidosis [38]. Previous methodological research has found that an adaptation period of 14 days was suitable for changeover and crossover nutritional experiments with cattle fed forage-based diets [13]. Our experimental animals had adapted for four months on a pre-experimental diet (Table 1) before the start of experiment. Therefore, there was a very low chance that insufficient adaptation was offered before the experiment which could cause the discrepancy.

Previous studies on the adaptation period focused on monitoring the variation in feed intake, the composition of gastrointestinal contents and feces and rumen fermentation indexes [13]. It has been reported that Holstein cows on the same diet showed similar volatile fatty acid content, pH and production performance, but different rumen bacterial community composition [39]. Palmonari et al. (2010) reported that similar bacterial composition also showed variable pH in ruminally cannulated cows. These studies indicated that the similarity of rumen fermentation or performance was not necessarily in accordance with similar bacterial community composition. Therefore, the time required for cattle to adapt well to a changed diet was quite variable and ranged from two days to 14 days based on different assessment indicators [13,37]. A valid approach to assessing the relative abundances of microbiota constituents at different taxonomic levels is provided by 16S rRNA gene sequencing [40]. Adopting this standard technique, many studies have explored the resilience and re-establishment of a bacterial community due to diet shifts on cannulated cattle [6,10]. However, the results obtained from cannulated cattle may be different from cattle under normal feeding conditions, because fistulated cattle generally have a higher concentration of oxygen in the rumen due to the installation of the cannulation and sampling exposure to the air [41]. In the current study, 16S rRNA gene sequencing was used to evaluate the monthly variation in the bacterial community of non-cannulated finishing steers. 

As the most abundant phyla in both diet groups, *Bacteroidetes* and *Firmicutes* did not show significant differences between the time intervals of months. However, the abundance of *Prevotellaceae_UCG-003*, a genus belonging to the phylum of *Bacteroidetes*, showed differences between C1 and C2 in the C diet group. A previous study suggested that some changes could occur at the class or genus levels despite no statistical significance of the rumen microbiota at the phylum level [13]. Thus, this could be a possible explanation for the differences that appeared at the genus level in the present study due to sampling intervals. In that way, we guessed that a longer time was required to achieve a relative stable microbial community at all levels.

### 4.4. Rumen Ecosystem Diversity as Time Advances

The rumen ecosystem diversity increases with age after birth [42,43]. Earlier studies in calves revealed that aerobic and facultative anaerobic microbiota constituents accounted for a large proportion before six weeks of age and were gradually replaced by anaerobic taxa until reaching a constant level [44]. In terms of specific phyla, *Bacteroidetes* increased rapidly after birth and became the most predominant phylum from two months to two years of age; *Proteobacteria* occupied a large proportion in newborn calves, whereas it accounted for a small percentage after six months of age [43]. Another study in pre-ruminant calves showed that *Bacteroidetes* dominated nearly three-quarters of the rumen microbiota at the age of six weeks, whereas it occupied less than half at two weeks of age [45]. The present study showed that *Bacteroidetes* and *Firmicutes* were the two most abundant phyla in both diet groups, displaying 66.44% and 25.16%, and 67.19% and 21.13% relative abundances in the C and L diet groups, respectively. The addition of monensin could explain the relative lower abundance of *Firmicutes* than that in previous reports [22,46] because monensin has selective inhibition to gram-positive bacteria [47]. The relative abundance of *Proteobacteria* increased from 0.70% in C1 to 5.33% in C3 in this study, and this may be due to the higher starch content (34.8% vs. 30.4%) in the C diet than that in the pre-experimental diet because this phylum was regarded as a core microbiota in digesting soluble carbohydrates. A small but important bacterial phylum for ruminant animals, *Fibrobacteres*, that is capable of degrading and digesting plant-based cellulose has attracted much attention in the past decade due to rising interest in the gastrointestinal microbiota [48]. In our study, *Fibrobacteres* increased as months advanced in the L diet group and reached at a peak in the second month in the C diet group. It is well known that cellulolytic bacterial abundances increase as the ratio of concentrate to forage decreases, whereas amylolytic bacterial abundances increase as the proportion of dietary concentrate increases [37,49]. It seemed that *Fibrobacteres* acclimated earlier in the C diet group, where a higher proportion of concentrate was offered in the C diet as compared to the L diet, which is consistent with the study of Anderson et al. (2016). These differences between the C and L diets may be due to the responsive capacity to the types of dietary transition, for example, transferring to higher ME (2.44 vs. 2.53) in the C diet and lower ME (2.44 vs. 2.35) in the L diet. The variations observed here provide a deeper insight into the microbial adaptability to the types of dietary transition.

Differences at the genus level may explain changes in microbiological development in a more detailed way as fattening months advanced. The present results showed that L1 accounted for the higher abundance of *Rikenellaceae_RC9_gut_group* than that in L2 and L3; this phenomenon could be interpreted as a result of the higher content of starch in the pre-experimental diet than that in the L diet (30.4% vs. 25.9%, respectively) because the family *Rikenellaceae* is found to be involved in the degradation of structural carbohydrates [50]. This indicated that a time-dependent effect on some bacteria before stabilization may occur as the months progressed. A previous study has found that *Fibrobacteraceae* and *Ruminococcaceae* are responsible for the degradation of cellulose [51]. Our results showed that *Ruminococcaceae_NK4A214_group* accounted for a high proportion in the first month and *Fibrobacter* had a high relative abundance in the last two months in both the two diet groups, which indicated that subtle differences in the composition of cellulolytic bacteria may occur as the months advance but may reach a relatively balanced population for fiber degradation (e.g., a higher proportion of *Ruminococcaceae* in C1 and a higher proportion of *Fibrobacteraceae* in C2 and C3 to contribute to cellulose degradation, Figure S3). *Eubacterium_coprostanoligenes* is reported to have the function of reducing cholesterol content and was found to have a higher relative abundance in high-yield dairy cows [52,53]. The continuous declination was observed in abundance in *Eubacterium_coprostanoligenes_group* as months advanced in both diet groups, but only with significant differences between L1 and L2 or between L1 and L3 along with no interaction between diet and period. *Ruminobacter* was found to be strongly responsive to dietary transition and was widely observed in a high-grain diet [31,54]. Therefore, it was expected that *Ruminobacter* abundance increased with the passage of time in the C diet because of adaptation to a higher starch content diet.

Recently, monitoring of the rumen bacterial diversity of buffaloes from birth to one year old revealed that the rumen microbial population altered with age even after six months of age [55]. Admittedly, the animals selected in the current study were used for fattening, and so naturally body weight (BW) and feed intake increased as fattening time advanced. Jami et al. (2013) observed an age-dependent increase in bacterial diversity; however, we observed similarities in bacterial diversity in both the C and L diet groups. This discrepancy indicates that small fluctuations in BW or feed intake had little effect on the bacterial diversity of cattle over 15 months old, from which it could be interpreted that bacterial diversity was associated with feed efficiency but not feed intake [4,5].

## 5. Conclusions

This study tracked the monthly dynamic variation in rumen bacterial community composition of finishing steers after a shift to a new diet. Our results showed that, despite similarities in the richness and evenness of bacterial community composition, some significant differences at the phylum and genus levels did exist among monthly sampling intervals. Most of the differences occurred between sampling in the first month and last two months, but still some phyla and genera were in temporal dynamics, indicating that the ruminal bacterial community does not stabilize even after an adaptation period of three months on the same diet. Moreover, the differences in the abundances of phyla and genera between monthly sampling intervals were affected by diets. These results provide insight into the combinations of diversity and certain taxa for assessing the adaptation of microbiota in dietary transition. A track study in longer time and various diets will be beneficial to confirm the stabilization time of a bacterial community and then provide guidance to sampling frequency and diet optimization.

## Figures and Tables

**Table 1:** Ingredients and nutrient composition of the experimental and pre-experimental diets.

Item		Diets ^1^		
		C	L	P
Ingredients, % of DM	Corn	41.44	29.94	35.69
	Wheat	5.73	4.14	4.94
	Soybean meal	7.37	5.32	6.34
	*Leymus chinensis*	43.78	59.38	51.58
	Calcium carbonate	0.56	0.41	0.48
	Sodium chloride	0.56	0.40	0.48
	Vitamin-mineral premix ^2^	0.56	0.41	0.49
Nutrient composition, % of DM	Metabolizable energy (ME), Mcal/kg	2.53	2.35	2.44
	Crude protein (CP)	11.9	10.5	11.2
	Neutral detergent fiber (NDF)	37.1	46.4	41.8
	Acid detergent fiber (ADF)	20.2	25.8	23.0
	Starch	34.8	25.9	30.4
	Calcium	0.48	0.50	0.49
	Phosphorous	0.26	0.23	0.25

^1^ C = standard energy and standard protein diet, L = low energy and low protein diet, P = pre-experimental diet. ^2^ Manufactured by Tangshan Mahanen Feed Co., Ltd., Tangshan, Hebei, China. Premix provided the following per kg of dry matter (DM): 5000 IU of vitamin A, 3000 IU of vitamin D3, 45 mg of vitamin E, 30 mg of monensin, 60 mg of Fe, 63 mg of Zn, 99 mg of Mn, 200 mg of Cu, 0.5 mg of Se, 1.1 mg of I, 0.45 mg of Co, 877.4 g of rice bran.

**Table 2:** The number of operational taxonomic units (OTUs), mean richness and evenness estimates for rumen samples collected at three monthly intervals in two diet groups.

Item ^1^	C ^2^				L ^3^				SEM ^4^	*p*-Value ^5^		
	C1	C2	C3	Mean	L1	L2	L3	Mean		Diet	Period	Diet × Period
OTUs	1248	1354	1379	1327	1530	1686	1414	1543	73.08	0.023	0.227	0.192
Chao1	1658	1866	1832	1786	1998	1888	2355	2080	124.9	0.064	0.199	0.247
Observed species	1204	1330	1306	1280	1478	1363	1622	1488	69.11	0.021	0.225	0.182
PD whole tree	93.51	99.07	98.30	96.96	107.5	101.1	114.6	107.7	4.076	0.034	0.309	0.275
Shannon index	7.816	7.996	8.121	7.978	8.340	7.995	8.419	8.251	0.139	0.095	0.092	0.118

^1^ OTUs, operational taxonomic units; PD whole tree, phylogenetic diversity whole tree. ^2^ C = standard energy and standard protein, where C1 indicates the first month in the C diet group and C2 indicates the second month in the C diet group, and the same pattern for C3; mean indicates the average value of C1, C2 and C3. ^3^ L = low energy and low protein, where L1 indicates the first month in the L diet group and L2 indicates the second month in the L diet group, and the same pattern for L3; mean indicates the average value of L1, L2 and L3. ^4^ SEM = standard error of means. ^5^ Diet = the effect of diets. Period = the effect of periods. Diet × Period = the interaction between diet and period; lowercase letters are marked only when the effect of periods was significant.

**Table 3:** Phylum (relative abundance > 0.5%) composition for rumen samples collected at three monthly intervals in two diet groups.

Phylum Name	C ^1^				L ^2^				SEM ^3^	*p*-Value ^4^		
	C1	C2	C3	Mean	L1	L2	L3	Mean		Diet	Period	D × P
*Bacteroidetes*	68.04	69.36	61.90	66.44	67.71	69.95	65.43	67.69	2.707	0.579	0.128	0.717
*Firmicutes*	27.33	22.35	25.80	25.16	23.64	19.00	20.76	21.13	2.795	0.057	0.311	0.913
*Proteobacteria*	0.702 ^b^	1.788 ^ab^	5.332 ^a^	2.607	1.608	2.718	2.947	2.424	0.785	0.818	0.034	0.168
*Fibrobacteres*	0.560 ^b^	1.505 ^a^	1.245 ^ab^	1.103	1.284 ^b^	2.302 ^ab^	3.569 ^a^	2.385	0.349	0.002	0.008	0.112
*Kiritimatiellaeota*	0.832	1.204	1.485	1.174	1.842	1.514	2.334	1.897	0.469	0.180	0.402	0.702
*Spirochaetes*	0.723	0.953	0.887	0.854	1.085	1.050	1.100	1.078	0.199	0.197	0.851	0.791
*Cyanobacteria*	0.138 ^b^	0.751 ^a^	0.903 ^a^	0.597	0.833	1.304	1.828	1.322	0.222	0.009	0.008	0.726
*Tenericutes*	0.398	0.674	0.916	0.663	0.577	0.658	0.464	0.566	0.095	0.170	0.134	0.024
*Patescibacteria*	0.479	0.665	0.551	0.565	0.485	0.546	0.540	0.524	0.083	0.452	0.402	0.712

^1^ C = standard energy and standard protein, where C1 indicates the first month in the C diet group and C2 indicates the second month in the C diet group, and the same pattern for C3; mean indicates the average value of C1, C2 and C3. ^2^ L = low energy and low protein, where L1 indicates the first month in the L diet group and L2 indicates the second month in the L diet group, and the same pattern for L3; mean indicates the average value of L1, L2 and L3. ^3^ SEM = standard error of means. ^4^ Diet = the effect of diets. Period = the effect of periods. D × P = the interaction between diet and period; lowercase letters are marked only when the effect of periods was significant; different lowercase letters (“a” or “b”) within the same row indicate differences, “ab” indicates both similarities with “a” and “b”.

**Table 4:** Genus (relative abundance > 0.5%) composition for rumen samples collected at three monthly intervals in two diet groups.

Genus Name	C ^1^				L ^2^				SEM ^3^	*p*-Value ^4^		
	C1	C2	C3	Mean	L1	L2	L3	Mean		Diet	Period	D × P
*Prevotella*	33.29	32.94	29.07	31.77	23.45	34.16	30.80	29.47	4.337	0.475	0.531	0.391
*Rikenellaceae_RC9_gut_group*	10.20	7.27	6.41	7.96	16.21 ^a^	9.12 ^b^	7.54 ^b^	10.95	1.236	0.021	0.001	0.151
*Prevotellaceae_UCG-003*	2.008	3.884	3.393	3.095	4.677	4.417	4.764	4.619	0.690	0.062	0.372	0.270
*Succiniclasticum*	3.414	2.588	3.232	3.078	2.111	1.392	1.870	1.791	0.445	0.004	0.279	0.979
*Prevotellaceae_UCG-001*	1.682	3.124	1.738	2.181	1.674	1.852	1.783	1.770	0.417	0.402	0.276	0.363
*uncultured_bacterium*	1.854	2.665	1.783	2.101	1.670	1.576	1.652	1.633	0.390	0.166	0.548	0.428
*Ruminococcaceae_NK4A214_group*	3.127 ^a^	1.433 ^b^	2.040 ^ab^	2.200	1.876 ^a^	1.169 ^b^	1.095 ^b^	1.380	0.261	<0.001	0.025	0.318
*Fibrobacter*	0.552 ^b^	1.500 ^a^	1.243 ^ab^	1.098	1.273 ^b^	2.299 ^ab^	3.565 ^a^	2.379	0.348	0.002	0.008	0.111
*Ruminococcus_2*	1.759	1.486	1.874	1.706	0.959	0.999	0.437	0.798	0.311	0.014	0.765	0.277
*Christensenellaceae_R-7_group*	1.386	1.214	1.301	1.300	1.156	0.923	1.139	1.073	0.234	0.137	0.686	0.948
*Ruminococcaceae_UCG-011*	1.149	1.505	1.069	1.241	1.250	0.991	1.076	1.106	0.259	0.572	0.794	0.478
*Prevotellaceae_UCG-004*	0.984	1.310	1.267	1.187	0.613	1.385	0.965	0.988	0.307	0.499	0.252	0.660
*Ruminococcaceae_UCG-014*	0.549	0.842	1.295	0.895	1.055	1.043	0.727	0.942	0.197	0.823	0.480	0.043
*Succinivibrionaceae_UCG-002*	0.105	0.493	2.534	1.044	0.579	0.837	0.948	0.788	0.508	0.623	0.106	0.204
*Treponema*	0.654	0.860	0.789	0.768	0.962	0.932	0.970	0.955	0.195	0.266	0.873	0.816
*Prevotellaceae_NK3B31_group*	1.227	0.692	0.897	0.939	0.645	0.277	0.733	0.552	0.271	0.216	0.276	0.721
*Eubacterium_coprostanoligenes_group*	1.079	0.689	0.573	0.780	0.914 ^a^	0.501 ^b^	0.441 ^b^	0.619	0.120	0.141	0.013	0.907
*Veillonellaceae_UCG-001*	0.952	0.493	0.544	0.663	0.774	0.537	0.798	0.703	0.132	0.707	0.119	0.362
*Ruminococcus_1*	0.400	0.675	0.882	0.652	0.822	0.520	0.728	0.690	0.162	0.794	0.399	0.205
*Lachnospiraceae_NK3A20_group*	0.752	0.786	0.871	0.803	0.449	0.510	0.565	0.508	0.127	0.043	0.542	0.967
*Selenomonas*	0.615	0.634	0.869	0.706	0.465	0.723	0.568	0.585	0.119	0.309	0.341	0.312
*Ruminococcaceae_UCG-010*	0.659	0.647	0.455	0.587	0.998 ^a^	0.509 ^b^	0.524 ^b^	0.677	0.116	0.361	0.040	0.177
*Saccharofermentans*	0.934	0.549	0.536	0.673	0.789	0.505	0.459	0.584	0.127	0.354	0.053	0.903
*Moryella*	0.819	0.827	0.935	0.860	0.589	0.248	0.289	0.375	0.124	0.004	0.374	0.188
*unidentified_rumen_bacterium_RFN46*	0.261	1.766	0.301	0.776	0.244	0.148	0.710	0.367	0.315	0.197	0.211	0.067
*CAG-352*	0.385	0.684	0.875	0.648	0.520	0.552	0.285	0.452	0.149	0.175	0.495	0.071
*Candidatus_Saccharimonas*	0.470	0.633	0.525	0.543	0.446	0.513	0.527	0.495	0.081	0.388	0.429	0.714
*Ruminobacter*	0.027 ^b^	0.364 ^ab^	0.985 ^a^	0.459	0.270	0.700	0.692	0.554	0.166	0.536	0.023	0.243

^1^ C = standard energy and standard protein, where C1 indicates the first month in the C diet group and C2 indicates the second month in C diet group, and the same pattern for C3; mean indicates the average value of C1, C2 and C3. ^2^ L = low energy and low protein, where L1 indicates the first month in the L diet group and L2 indicates the second month in the L diet group, and the same pattern for L3; mean indicates the average value of L1, L2 and L3. ^3^ SEM = standard error of means. ^4^ Diet = the effect of diets. Period = the effect of periods. D × P = the interaction between diet and period; lowercase letters are marked only when the effect of periods was significant; different lowercase letters (“a” or “b”) within the same row indicate differences, “ab” indicates both similarities with “a” and “b”.

## Supplementary Materials

The following are available online at https://www.mdpi.com/2076-2607/7/10/410/s1.
