# Supplementary material for: Temporal Dynamics in Rumen Bacterial Community Composition of Finishing Steers during an Adaptation Period of Three Months
Source: Microorganisms. 2019 Oct 1;7(10):410. doi: 10.3390/microorganisms7100410 (PMC6843415; doi:10.3390/microorganisms7100410)
Supplement: Supplementary file 1 [file microorganisms-07-00410-s001.pdf]

Supplementary Materials

Table S1. Basic sample and sequencing information

Sample ID [1]	Steers ID	Raw reads	Quality filtered reads	Ambiguous bases
C11	S1682	65508	64884	124
C12	S1603	108582	107328	229
C13	S1638	102092	101198	235
C14	S1662	102802	101288	230
C15	S1661	91512	90310	174
L11	S1689	102180	101274	242
L12	S1635	112804	111474	234
L13	S1627	116848	115712	298
L14	S1626	103056	102140	292
L15	S1675	58404	57704	114
C21	S1682	50894	50318	121
C22	S1603	84512	83516	143
C23	S1638	70058	69318	179
C24	S1662	52530	51852	145
C25	S1661	46358	46000	64
L21	S1689	48866	48496	70
L22	S1635	104894	104214	237
L23	S1627	51724	51264	91
L24	S1626	51572	51090	106
L25	S1675	80518	79648	201
C31	S1682	43400	43092	86
C32	S1603	117544	116632	238
C33	S1638	83284	82094	162
C34	S1662	108064	106238	290
C35	S1661	50712	50136	121
L31	S1689	46040	45794	131
L32	S1635	54156	53848	96
L33	S1627	80904	80112	188
L34	S1626	141964	138864	36
L35	S1675	84268	83312	209

[1] C = standard energy and standard protein diet, C11 indicates the first animal in the first month of C diet, C21 indicates the first animal in the second month of C diet, C31 indicates the first animal in the third month of C diet, the same pattern for other Sample ID in C diet; L = low energy and low protein diet, L11 indicates the first animal in the first month of L diet, L21 indicates the first animal in the second month of L diet, L31 indicates the first animal in the third month of L diet, the same pattern for other Sample ID in L diet.

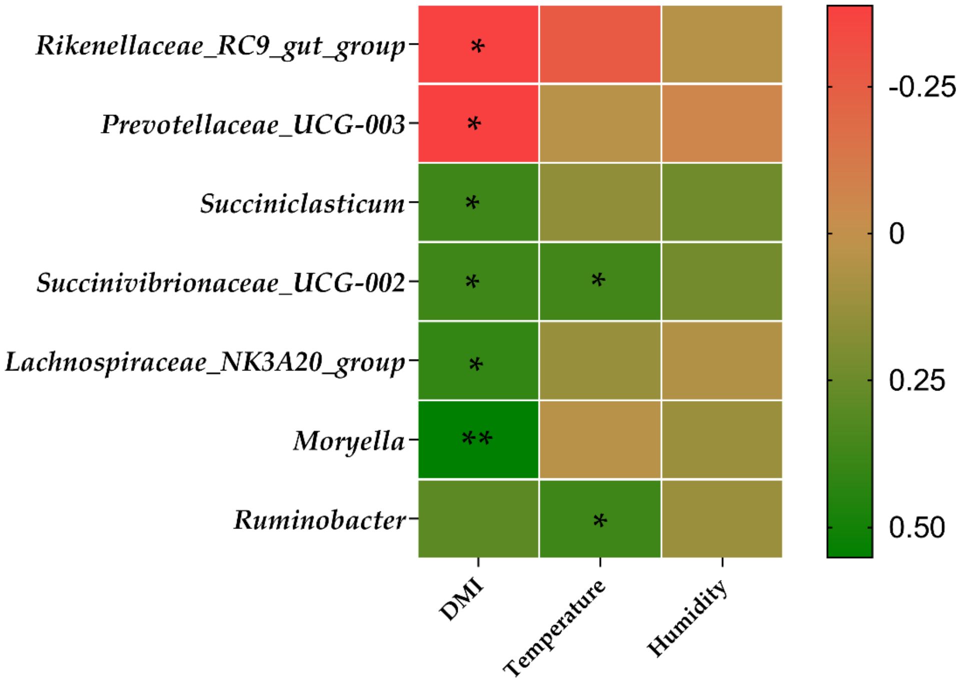

Figure S1: Correlations between rumen genera and DMI, temperature and humidity. One asterisk (*) within each cell indicates $0.01 \le p < 0.05$, two asterisks (**) within each cell indicate $p < 0.01$, and no asterisk within each cell indicates $p \ge 0.05$.

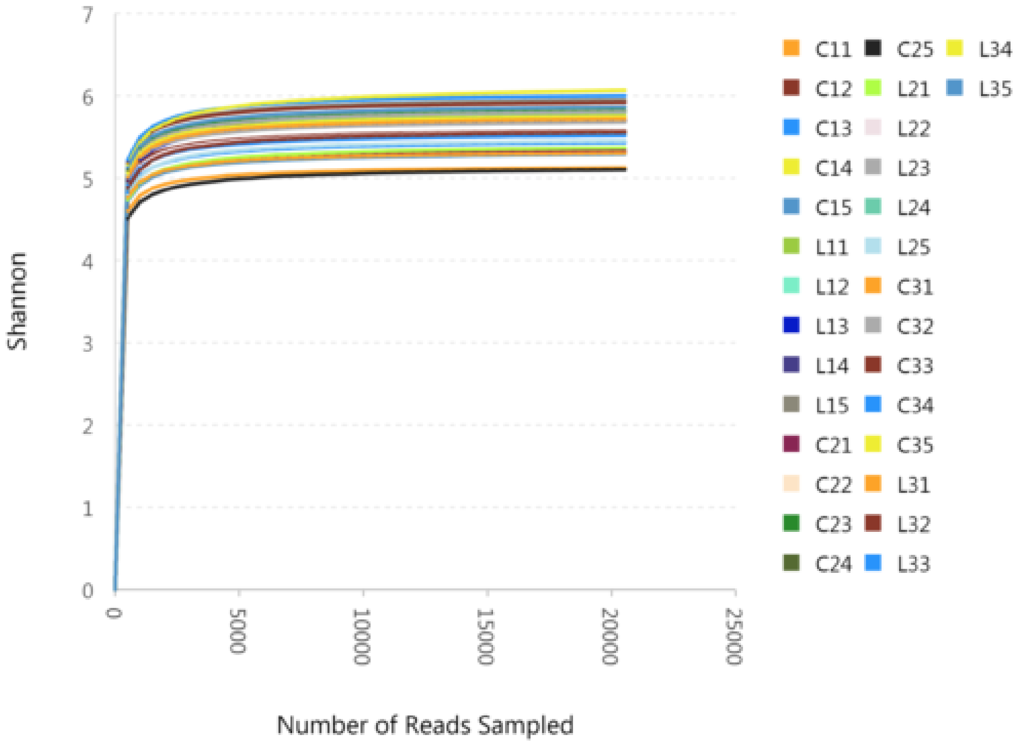

Figure S2: Shannon–Wiener curves based on Shannon index for standard energy and standard protein diet (C) and low energy and low protein diet (L).

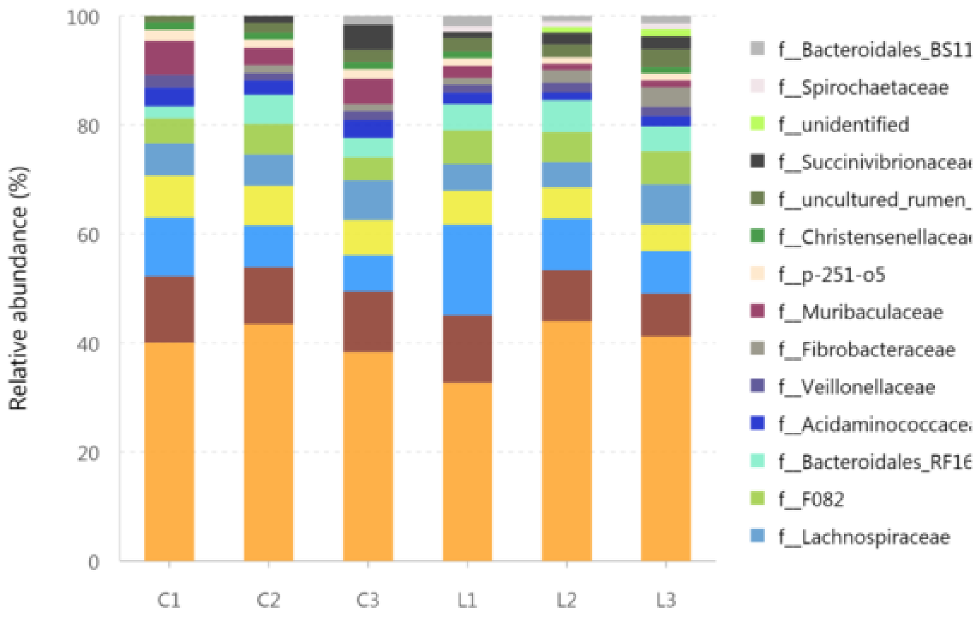

Figure S3: Relative abundance at family level in standard energy and standard protein diet (C) and low energy and low protein diet (L). C1 indicates rumen samples collected from the first month in C diet, C2 indicates rumen samples collected from the second month in C diet, and C3 indicates rumen samples collected from the third month in C diet, the same pattern is for L diet.
